# Virus–Host Coevolution with a Focus on Animal and Human DNA Viruses

**DOI:** 10.1007/s00239-019-09913-4

**Published:** 2019-10-10

**Authors:** Győző L. Kaján, Andor Doszpoly, Zoltán László Tarján, Márton Z. Vidovszky, Tibor Papp

**Affiliations:** Institute for Veterinary Medical Research, Centre for Agricultural Research, Hungarian Academy of Sciences, Hungária krt. 21, Budapest, 1143 Hungary

**Keywords:** Virus, Coevolution, Antiviral defence, Endogenous viral elements

## Abstract

Viruses have been infecting their host cells since the dawn of life, and this extremely long-term coevolution gave rise to some surprising consequences for the entire tree of life. It is hypothesised that viruses might have contributed to the formation of the first cellular life form, or that even the eukaryotic cell nucleus originates from an infection by a coated virus. The continuous struggle between viruses and their hosts to maintain at least a constant fitness level led to the development of an unceasing arms race, where weapons are often shuttled between the participants. In this literature review we try to give a short insight into some general consequences or traits of virus–host coevolution, and after this we zoom in to the viral clades of adenoviruses, herpesviruses, nucleo-cytoplasmic large DNA viruses, polyomaviruses and, finally, circoviruses.

## Coevolution in General

Viruses are obligatory cellular parasites, developing with their hosts since the dawn of life. Coevolution, when the virus and the host reciprocally affect each other’s evolution, is often detected. According to the Red Queen Hypothesis, both the parasite and the host are perpetually struggling to maintain a constant fitness level (McLaughlin and Malik 2017). This long-term evolutionary pressure gave rise to some surprising consequences for the entire tree of life.

## The Origin of Viruses—Which Came First: The Chicken or the Egg?

What can be the origin of an obligatory cellular parasite? Three concurring hypotheses describe the origin of viruses: (i) the primordial virus world or the virus first hypothesis claims that the ancestors of viruses existed already in the pre-cellular world; (ii) the escaped genes theory describes viruses as mobile genetic elements, which became independent of their host cells; (iii) whereas according to the cellular regression theory, viruses are regressed intracellular parasites (Holmes 2013).

### The Primordial Virus World

According to the primordial virus world hypothesis, in the primordial soup multiple pre-cellular and pre-viral Ur-organisms competed with each other. The last universal common ancestor—the first cellular life form—emerged from these primordial replicators, and at least a fraction of current viruses might originate from the remaining ones (Moelling and Broecker 2019). Ancient viruses might have even contributed to the formation of the last universal common ancestor of all living organisms.

It is generally accepted that an RNA-based world preceded today’s DNA- and protein-based one. RNA viruses and especially the capsidless viroids might resemble this ancient world (Elena et al. 1991). However, the evolution of viruses—which has been especially rapid for RNA viruses—destroyed the possible signal of genetic relatedness a long time ago, making phylogenetic reconstruction already impossible. Luckily, some sort of evidence is still within reach: the existence of viral protein fold superfamilies without cellular counterparts. Proteins without any primary sequence homology are still grouped based on their conserved tertiary structure, their folding: e.g. the major capsid proteins of the PRD1 bacteriophage, adenoviruses and some archaeal viruses all have a double jelly-roll fold structure (Benson et al. 1999; Nasir and Caetano-Anollés 2015, 2017), or there are structural homologies in the RNA- and DNA-dependent polymerases too (Gorbalenya et al. 2002). There are fold superfamilies containing both viral and cellular proteins (we will return to this phenomenon by the escaped genes theory), but there are also numerous examples without cellular homologues found in phylogenetically very diverse viruses: e.g. viral RNA-dependent RNA polymerases are not homologous to their cellular counterparts (Iyer et al. 2003). According to the most parsimonious assumption, these proteins have a common origin in the primordial virus world, and phylogenetic tree reconstructions of these fold superfamilies confirm this as well (Nasir and Caetano-Anollés 2015).

Viruses became obligatory parasites either only after the emergence of the last universal common ancestor, or it is equally possible that they were already parasitizing on other ancient replicators. According to the viral eukaryogenesis hypothesis, even the eukaryotic cell nucleus originates from an infection by a coated virus: the virus became an endosymbiont usurping the role of the original archaean nucleus (Bell 2009). The endosymbiont pox-like virus had no possibility for a lytic viral cycle, and under this evolutionary pressure started to replicate by means of cell-to-cell fusion using its fusion proteins. When two such cells—infected by related viruses—fused to each other, the viral DNA was copied, and homologous viral chromosomes formed tetrads. Cell division resulted in four daughter cells with one copy of the endosymbiont virus in each. This might be the origin of meiotic cell division (Bell 2006). After its development, sexual reproduction offered a significant fitness advantage in the fight against any parasite (Hamilton et al. 1990), including viruses.

### Escaped Genes

This theory states that mobile genetic elements escaped the cell, acquired a protein capsid and started to replicate autonomously (Moreira and López-García 2005, 2009). Numerous homologies can be observed among viral and cellular genes. On the other hand, the root position, and thus the certain direction of evolution, might be challenging to determine. For example, cellular and viral DNA polymerases are related (Filée et al. 2002), but the exact direction of the lateral gene transfer is unknown (Shackelton and Holmes 2004).

### The Cellular Regression Theory

Perhaps the least favoured theory currently claims that viruses are the extremely reduced descendants of obligatory intracellular parasites, incapable of autonomous extracellular life (Bândea 1983). According to this theory, the viral genome is the remnant of a heavily reduced cellular genome and the capsid is that of a cell membrane. The nucleo-cytoplasmic large DNA viruses (NCLDVs) are good candidates to represent the end result of this mechanism: they proliferate in the cytoplasm, and have particle and genome sizes comparable to those of the smallest prokaryotes (Arslan et al. 2011; Colson et al. 2012, 2011). The modern version of this theory was mentioned when discussing the primordial virus world: viruses might be the reduced descendants of pre-cellular Ur-organisms, converting into obligatory cellular parasites (Claverie 2006; Forterre 1991).

## Viral Genetic Elements in Host Genomes

### Polintons

The theories of viral origin are a topic of heated debate, but most possibly none of them is mutually exclusive, and the suspected processes of two or more could have occurred parallel and/or sequentially as well. A small proportion of mobile genetic elements might have its root in the primordial virus world, but numerous regression or escape occasions must have occurred too. The latter were perhaps facilitated by the cellular genomic insertion of viral genetic elements. Polintons, also known as Mavericks, are mobile genetic elements with multiple homologues in both cellular and viral genomes. They have escaped several times and gave rise to different viruses and mobile genetic elements such as adenoviruses or mitochondrial linear plasmids (Krupovic and Koonin 2015, 2017). It must be stressed again that these genetic elements are homologous and related, but the direction of the genetic exchange is unknown in most cases. Such transfers are common from host to virus and vice versa too.

### Endogenous Viral Elements

Endogenous viral elements (EVEs) are viral genes or sometimes complete genomes inserted into host genomes. As genome integration is a compulsory step in retroviral replication, most of these elements are of retroviral origin, but further virus families were detected as well: hepadnaviruses, adeno-associated viruses, herpesviruses and others (Aiewsakun and Katzourakis 2015; Bill and Summers 2004; Broecker and Moelling 2019; Morissette and Flamand 2010; Young and Samulski 2001). If the insertion occurs in the germ cell line, the inserted sequence stretch might get inherited. And if it provides a selective advantage, e.g. protection from a viral infection, the genomic change will be fixed in the population. In case this advantage disappears, the inserted stretch might mutate over time and lose its protective nature (Broecker et al. 2016).

EVEs are important sources for virus–host coevolutionary research, as they provide a map for viral host range. The history of coevolution can be traced by the detection of homologous endogenous viral sequences in different host species. This provides information about the historical host range of a virus clade, and even the time frame of coevolution can be inferred based on the known diversification times of the hosts, or on phylogenetic analyses of exo- and endogenous viral sequences. E.g. based on endogenous lentiviral sequences in lemurs, it was hypothesised that these viruses have been coevolving with primates for several millions of years (Katzourakis et al. 2007).

Viruses represent a major force in evolutionary pressure (Emerman and Malik 2010; Villarreal and Witzany 2010). Retroviruses, for example, are so effective in genome integration and have been coevolving with vertebrates so long, that over 50% of the human genome was estimated to consist of endogenous retroviral elements (de Koning et al. 2011). Some of these regions provide important and fundamental attributes to their integrator hosts, like the syncytin genes, which have an important role in placental development not only in various mammals but in viviparous lizards as well (Cornelis et al. 2017; Imakawa and Nakagawa 2017). Other transposable elements contribute to embryonic development, stem cell pluripotency or cell differentiation in eukaryotes (Chuong et al. 2017). In prokaryotes, important functions were attributed to the integrated viruses as well. E.g. in *E. coli*, deleting all prophages has resulted in increased susceptibility to environmental factors and slower cell growth (Wang et al. 2010).

## Effects of the Bottleneck Phenomenon

Essentially, the viral mutation rate and copy number determine the variability within the host and *in fine* the stock of viral evolution (Duffy et al. 2008; Peck and Lauring 2018; Sanjuán and Domingo-Calap 2016). Generally, viral pathogens exist in diverse populations in vivo (McCrone and Lauring 2018). As a substantial evolutionary effect on the population dynamics of viruses, genetic bottleneck (reduction of effective population size due to environmental events, for instance new habitat colonisation) affects the virus–host coevolution: it decreases both the genetic variation and the fitness of the virus, and furthermore it is responsible for the founder effect (Novella et al. 1995). Changes of genotype frequencies by stochastic population size reduction are referred to as genetic drift (Bergstrom et al. 1999; Dennehy et al. 2006; Elena et al. 2001; McCrone and Lauring 2018; Zwart and Elena 2015). Furthermore, it is important to stress that the phenomenon of genetic bottleneck is interpretable from both virus and host perspective (Voskarides et al. 2018).

In the case of the viruses, the newly colonised region might be a new host (host switch) or organ (Voskarides et al. 2018; Zwart and Elena 2015). Bottleneck events occur during both intra- and inter-species steps of the viral life cycle (Gutiérrez et al. 2012). An interesting aspect of virus evolution and the bottleneck effect is the case of multipartite viruses (e.g. plant-infecting nanoviruses or animal-infecting bidnaviruses, alphatetraviruses, nodaviruses and picobirnaviruses). These package their genetic material as independently encapsidated separate segments (Lucía-Sanz and Manrubia 2017). Self-evidently, bottleneck events have a crucial role for these viruses, as all genomic segments are needed for a successful infection. Furthermore, the different genomic segments drift at different rates (Gallet et al. 2018).

## Antiviral Defence Mechanisms

Under the constant threat of an infection, cellular organisms have developed multiple layers of different defence mechanisms (not solely) against these genetic parasites to protect themselves and their genomic integrity.

### Shuttled Weapons

Most strikingly, antiviral defence mechanisms often have viral origins too (Broecker and Moelling 2019; tenOever 2016; Villarreal 2009). The simplest mechanism is the superinfection exclusion, where an integrated and expressed endogenous viral protein provides protection from exogenous infection. This was first described in the tobacco plant, but it is applied by prokaryotes, animals or other plants as well (Moelling et al. 2017). The development of such a defence mechanism was observed in sheep and koalas for example. Retroviral elements were endogenised even as recently as 200 and 100 years ago in the genomes of sheep and koala, respectively, providing protection against certain exogenous retroviral infections (Armezzani et al. 2014; Tarlinton et al. 2006).

But such basic integrations are not the only examples. The recently described bacterial antiphage system, CRISPR-Cas (Charpentier and Doudna 2013), has its roots in at least five different classes of mobile genetic elements (Koonin and Makarova 2017). The Argonaute proteins show structural and functional homologies to the retroviral replication machinery and provide protection against invading nucleic acids in prokaryotes (Moelling et al. 2006). The RNA interference system of eukaryotes shares the same homologies, and it is available in each of the five eukaryotic superkingdoms, suggesting a very ancient evolutionary origin (Cerutti and Casas-Mollano 2006). Though present, this system does not provide antiviral effects in chordates. But also here, endogenous retroviruses provide transcription factor binding sites for interferon-stimulated genes (Chuong et al. 2016; Ito et al. 2017), whereas the Rag recombinases—needed for the diversification of antibodies—originate from transposons found in the genomes of starfish, oysters and sea urchins (Kapitonov and Koonin 2015).

### Other Mechanisms

The most ancient defence mechanism might be the use of antisense RNAs, where the gene translation of the invading pathogen is interfered by small complementary RNAs (Gottesman and Storz 2011). Against DNA phages, bacteria often use restriction endonucleases, cleaving the viral genome (Kobayashi 2001). Piwi-interacting RNAs associate with nucleases and provide defence against transposable elements. The latter are available in all metazoans, and they may have a common origin with the prokaryotic Argonaute system (Iwasaki et al. 2015). As already mentioned, the RNA interference does not provide an antiviral effect in chordates; instead, pattern recognition receptors were developed. Engagement of these by pathogen-associated molecular patterns (i) induces antiviral cytokines: the tumour necrosis factor, available in most eukaryotic lineages, but in chordates rather the interleukins and interferons (Levy et al. 2011), and (ii) activates the natural killer cells too (Esteso et al. 2017). The APOBEC3 protein is part of the innate immune system in humans, it targets specifically retroviruses and interferes with their reverse transcription (Sheehy et al. 2002). However, HIV counteracts this effect by the viral infectivity factor, which triggers the degradation of APOBEC3 (Donahue et al. 2008). Furthermore, it was also observed that human leucocyte antigen I and II loci are genetically more diversified in pathogen- (most predominantly virus-) rich environments (Prugnolle et al. 2005; Sanchez-Mazas et al. 2012).

After this short general introduction on the highlights of the topic, we focus on the coevolutionary processes of selected DNA viruses: adeno-, herpes-, NCLD-, polyoma- and circoviruses.

## Adenoviruses

Adenoviruses are DNA viruses, the major capsid proteins of the non-enveloped, icosahedral capsid are the hexon, the penton base and the protruding fibre, responsible for receptor binding. Their non-segmented, double-stranded, linear genome varies between 26 and 45 kbp in size, and the DNA is covalently bound to the terminal protein (Harrach 2014). Adenoviruses infect vertebrate hosts, and are clustered into five accepted and one proposed genera. Members of the genera *Mastadenovirus* and *Aviadenovirus* infect mammals and birds, respectively. Atadenoviruses were detected in reptiles, birds, ruminants and a marsupial possum; siadenoviruses in birds, a frog and a tortoise. The single member of the genus *Ichtadenovirus* is the white sturgeon adenovirus. The sixth genus was proposed recently: testadenoviruses were detected in testudinoid turtles only until now (Doszpoly et al. 2013).

As these viruses were described from five major classes of the vertebrates, the hypothesis was formed that adenoviruses had started to coevolve with the vertebrates 450 million years ago, before the divergence of fish from other vertebrates (Kovács et al. 2003). Mast-, avi-, at-, si- and ichtadenoviruses had been thought to coevolve with mammals, birds, reptiles, amphibians and fish, respectively (Benkő and Harrach 2003). This hypothesis was partially questioned later, as the white sturgeon and the frog adenovirus—the latter belonging to genus *Siadenovirus*—are the single fish or amphibian adenoviruses, respectively, discovered until now (Davison et al. 2000; Doszpoly et al. 2019). Furthermore, several avian adenoviruses clustered into the genus *Siadenovirus* (Ballmann and Harrach 2016; Kovács and Benko 2011; Kovács et al. 2010; Lee et al. 2014). Yet, it is equally possible that several further amphibian and fish adenoviruses are awaiting discovery.

Adenoviruses are generally thought to be host specific with usually one or very few host species for a specific viral serotype. Still, during the hundreds of millions of years, host changes did happen, e.g. there were ten predicted host switches in the evolution of human adenoviruses in 4.5 million years (Hoppe et al. 2015). The most striking assumed host switch happened for some atadenoviruses. These are thought to be the lineage coevolving with squamatid reptiles originally (Wellehan et al. 2004), but presumably a virus strain had jumped to an ancient ruminant during the evolutionary history. Atadenoviruses were isolated from cattle, sheep and mule deer, suggesting some level of coevolution already. However, the high genomic A + T content—hence the name atadenovirus—contradicts long cospeciation of these viruses (Benkő and Harrach 1998).

Non-reptile atadenovirus genomes are characterised by 57.0–66.3% A + T content, whereas reptile atadenoviruses are not affected by such bias (Farkas et al. 2002; Harrach 2008; Papp et al. 2009; Wellehan et al. 2004). Examples from other virus families are known: the influenzaviral nucleotide composition changes following a host jump from bird to mammal (Greenbaum et al. 2008), or similar differences were observed between flaviviruses or herpesviruses of different hosts (Jenkins et al. 2001; McGeoch et al. 2006). Obviously, this composition has an effect on codon usage, thus the complete genome sequence might be under evolutionary pressure (Shackelton et al. 2006). Still, the exact cause of this bias in atadenoviruses is unknown, but it is hypothesised that longer coevolutionary times—adaptation of the virus to the host—result in a balanced nucleotide composition (Farkas et al. 2002; Papp et al. 2009; Wellehan et al. 2004).

Similar observations were made about pathology. Adenoviruses generally cause a mild disease, and there are apathogenic strains at least in healthy individuals. E.g. recent research shows that only specific and not all serotypes of the species *Fowl aviadenovirus D* and *E* cause inclusion body hepatitis in chicken (Schachner et al. 2018; Zadravec et al. 2011). But recent host switches might result in an elevated pathogenicity (Benkő and Harrach 2003; Jánoska et al. 2011; Kohl et al. 2012; Vidovszky et al. 2015). For instance, duck adenovirus 1 is apathogenic in ducks and geese, but causes the egg drop syndrome in chickens (Hess et al. 1997). Another atadenovirus causes a haemorrhagic epizooty among mule deer (Lehmkuhl et al. 2001; Woods et al. 1996). Or bovine adenovirus 10—though a mastadenovirus—is only distantly related to other bovine mastadenoviruses, and it causes a severe acute fibrinous enterocolitis (Horner et al. 1989). The A + T content of the known genome part is over 59%, and from serologically identical strains several fibre size variants were described (Ursu et al. 2004). These mutations might be the consequences of an ongoing evolution, and represent the adaptation to a relatively new host, cattle (Benkő and Harrach 2003).

**Fig. 1:**
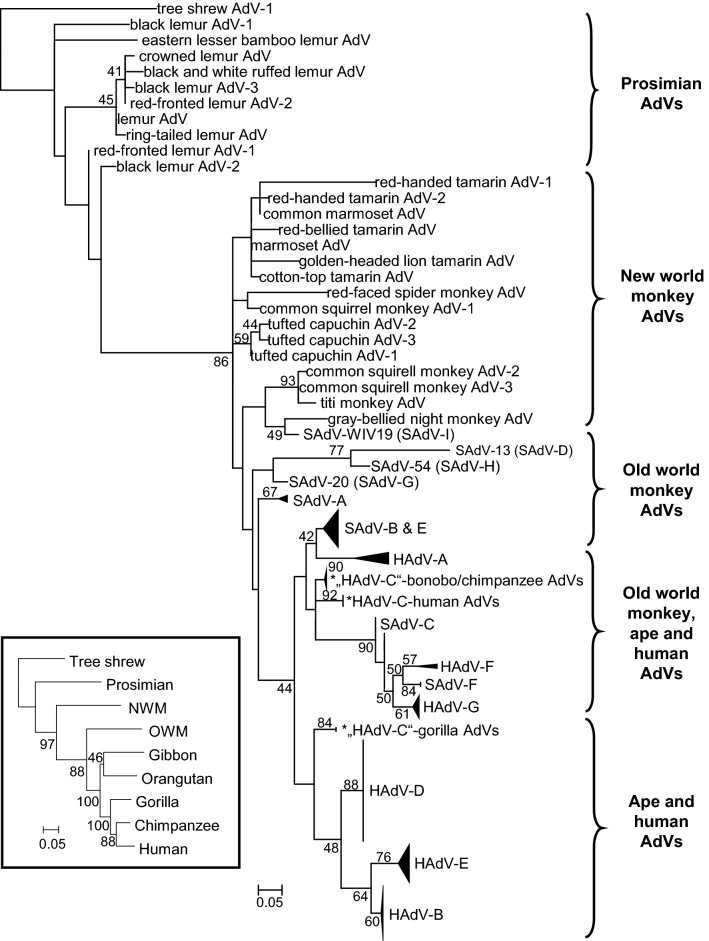
Phylogeny reconstruction (maximum likelihood analysis) based on partial sequences of the IVa2 protein of primate adenoviruses (AdVs). A phylogenetic tree of primates is shown in the bottom left corner to demonstrate the parallel evolution of AdVs and hosts. SAdV-A, *Simian mastadenovirus A*; HAdV-B, *Human mastadenovirus B*, etc. Hosts of AdVs presumably belonging to species HAdV-C are marked with asterisks due to their separation. [From Podgorski et al.: Adenoviruses of the most ancient primate lineages support the theory on virus–host coevolution (2018) Acta Vet Hung 66:474, with permission from Akadémiai Kiadó.]

The most investigated adenoviruses are evidently human and other primate adenoviruses. Codivergence is not obvious here at the first look as host switches blur the picture (Hoppe et al. 2015; Purkayastha et al. 2005; Roy et al. 2009; Wevers et al. 2011), but it is still observable. The most ancient lineages are the prosimian and New World monkey adenoviruses (Podgorski et al. 2018). On the next branches we can find the Old World monkey adenoviruses including members of *Human mastadenovirus* (HAdV) *A*, *F* and *G* (Gilson et al. 2016; Pantó et al. 2015; Podgorski et al. 2016) (Fig. 1). Evolutionarily speaking, strains of HAdV-B and -E are of gorilla or chimpanzee origin, respectively; and strains of HAdV-B jumped to the common ancestor of humans and chimpanzees two times most probably (Hoppe et al. 2015). HAdV-C seemingly codiverged with gorillas, chimpanzees, bonobos and humans, whereas HAdV-D viruses infect only humans (Hoppe et al. 2015). The long-term coevolution of HAdV-D and humans is supported by the high variety of types within the species (Ismail et al. 2018b; Kaján et al. 2018), and their usually facultative pathogen nature.

Exactly in HAdV-D strains it is often observed that homologous recombination has a driving force in adenovirus evolution (Crawford-Miksza and Schnurr 1996; Gonzalez et al. 2015; Ismail et al. 2018a; Kaján et al. 2017; Robinson et al. 2013; Walsh et al. 2009). Predominantly, intraspecies recombination events are common, but intergenus recombinants have also been reported already: the head domain of the porcine adenovirus 5 (genus *Mastadenovirus*) fibre seems to be of atadenoviral origin (Nagy et al. 2002), though a porcine atadenovirus has not been discovered yet. Recombination enables an accelerated, modular evolution of viruses, ‘and has been associated with such features as the evasion of host immunity (Malim and Emerman 2001), the development of antiviral resistance (Nora et al. 2007), the ability to infect new hosts (Hon et al. 2008), increases in virulence (Khatchikian et al. 1989) and even the creation of new viruses (Weaver 2006)’ (Holmes 2013).

## Herpesviruses

Herpesviruses (HVs) are large (150–200 nm) icosahedral, enveloped viruses having a so-called tegument layer between the capsid and the envelope. HVs possess a linear, non-segmented, double-stranded DNA genome (135–295 kbp) (Pellett et al. 2011). They are known to infect all classes of vertebrates, from fish to humans; moreover, HVs were discovered in some mollusc species as well (Davison et al. 2009). Chronologically, HVs of mammals and birds were known first causing different diseases in humans, livestock and poultry. Later, several decades ago, HVs of reptiles, fishes and amphibians were also reported (Fawcett 1956; Rebell et al. 1975; Wolf and Darlington 1971). These novel viruses were tentatively classified into the family *Herpesviridae* because of their morphological features. The first non-vertebrate HV was discovered in a mollusc species in the 1970s (Farley et al. 1972). As more sequence data became available, molecular analysis provided insight into the evolution of HVs, and these newly discovered viruses were also officially classified into the family *Herpesviridae* (Davison 1998; Davison et al. 2006, 2005; Davison and Davison 1995; McGeoch and Gatherer 2005). In 2009, the taxonomy of HVs changed radically, as the family *Herpesviridae* was split into three families. The novel family *Herpesviridae* contains only the HVs of higher vertebrates (Amniotes), while the family *Alloherpesviridae* contains the HVs of amphibians and fish (Anamnia), and the family *Malacoherpesviridae* contains the HVs of molluscs. The three families were clustered under the novel order *Herpesvirales* (Davison et al. 2009). These three groups of HVs are related only tenuously to each other. There are only a few genes showing homology in all known HVs, the most conserved gene is the putative ATPase subunit of terminase, its gene product being responsible for the packaging of the viral DNA into the capsid (Davison et al. 2009). Interestingly, a homologous gene was found in T4 bacteriophages (*Myoviridae*) implying the common origin of HVs and tailed bacteriophages (Baker et al. 2005).

The phylogenetic relationship of HVs is well studied and their evolution was found to be largely synchronous with host lineages (McGeoch et al. 2000, 2006). Exceptions have been noted among the mammalian (McGeoch et al. 2006) and fish HVs (Kelley et al. 2005). The major sublineages within the subfamilies (*Alpha-*, *Beta-*, *Gammaherpesvirinae*) of *Herpesviridae* emerged probably before the mammalian radiation, 60 to 80 million years ago, while the diversification time point of the three subfamilies had been around 200 million years ago (McGeoch et al. 1995, 2000). The most common ancestor of all known HVs (order *Herpesvirales*) existed around 400 million years ago based on similar phylogenetic branching patterns of HVs and host lineages (McGeoch et al. 2006).

As for mammalian HVs, several studies were conducted on their evolutionary route spanning shorter time periods. For example, *Bovine herpesvirus 4* (BoHV-4) has been isolated from cattle worldwide and this species was thought to be the original host of the virus. However, BoHV-4 has been reported from wild buffalo and other ruminants as well (Rossiter et al. 1989; Todd and Storz 1983). Later, serological studies implied that the original host of the virus might be the African buffalo (*Syncerus caffer*) (Dewals et al. 2005). This hypothesis was confirmed by phylogenetic calculations, suggesting that BoHV-4 has been coevolving with the African buffalo for the last 1.5 million years, and the virus was passed to cattle on at least three independent occasions much more recently, sometimes via an intermediate species (Dewals et al. 2006). In another study, the phylogenetic trees of primate cytomegaloviruses and their hosts’ were compared, and primate cytomegalovirus genomes were also analysed. The obtained results demonstrated the coevolution of these viruses with their hosts (Russell et al. 2016). Furthermore, the avian and reptilian HVs were clustered into the subfamily *Alphaherpesvirinae*, relating to the development of birds from reptilian progenitors (McGeoch and Gatherer 2005).

As for the members of the family *Alloherpesviridae*, phylogenetic inferences strongly support the monophyly of fish and amphibian HVs within the order *Herpesvirales* (Waltzek et al. 2009). The comparison of the phylogenetic trees of viruses and hosts implied that closely related HVs in the family may have coevolved with their hosts, whereas codiversification was not supported at deeper nodes of the tree (Waltzek et al. 2009). In other words, the separation of the main alloherpesvirus lineages does not fully resemble that of the host taxa. One explanation can be that the ancestors of the different viruses diverged before the separation of the various fish lineages, and thus the different virus lineages evolved independently. The strong similarity among viruses of distantly related fish species could be explained by host switches (Doszpoly et al. 2011).

Integrated herpesviral genomes in host chromosomes were also discovered in the last years, giving insight into the virus–host coevolution and providing interesting data about the genome organisation and content of ancient virus species (Aswad and Katzourakis 2017; Inoue et al. 2017; Savin et al. 2010). The discovery of a new lineage of alloherpesviruses associated with at least fifteen different fish species was reported. One of them seems to represent a full-length viral genome in salmon (*Salmo salar*) (Aswad and Katzourakis 2017). An interesting discovery of a HV genome integrated into the genome of an invertebrate chordate provides links to the ancient ancestors of mollusc and vertebrate HVs: the genome of *Branchiostoma floridae* (Florida lancelet or amphioxus) contains several genes showing homology to herpesviral genes, implying the existence of a HV associated with this invertebrate chordate. The terminase and polymerase gene sequences from the putative amphioxus HV show higher similarity to those of the mollusc HVs than to any vertebrate HVs (Savin et al. 2010). The discovery of this virus gave data about HVs dating back to the separation of vertebrates and invertebrates.

## Nucleo-cytoplasmic Large DNA Viruses

Nucleo-cytoplasmic large DNA viruses (NCLDV) are a group of DNA viruses including the families *Ascoviridae*, *Asfarviridae, Iridoviridae, Marseilleviridae*, *Mimiviridae*, *Pithoviridae*, *Phycodnaviridae* and *Poxviridae* (Koonin and Yutin 2010, 2019). A new virus order, the *Megavirales* was proposed [but not yet accepted by the International Committee on Taxonomy of Viruses (ICTV)] to collect the above-mentioned virus families (Colson et al. 2013). Their host range spans from unicellular eukaryotes via arthropods to vertebrates (even mammals). NCLDVs replicate within the cytoplasm of the infected cells, yet in some families (e.g. iridoviruses) a nuclear stage is also present. Hence, NCLDVs encode many genes required for their own successful replication, but still use the translational apparatus of the host. Although the genome size (between 100 and 2500 kb) and host range of NCLDVs vary greatly, they appeared to form a monophyletic group with a common ancestor, based on a subset of about 30 conserved genes (Filée et al. 2008). For instance, iridoviral homologues of ATPase, the A1L/VLTF2 transcription factor, the major capsid protein and the DNA polymerase proteins show considerable sequence similarity with asfar-, asco-, mimi-, pycodna- and poxvirus counterparts (Boyer et al. 2009).

In 2003, the discovery of mimivirus, the first giant amoeba virus (La Scola et al. 2003) with its large virion (700 nm) in the size range of bacteria and archaea, altered a century-long vision about the sizes and genome structures in the virosphere. Its genome was also huge (1200 kb) (Raoult et al. 2004), comparable to the smallest free-living prokaryote genomes (Koonin 2009), and contained several genes of distant origins (eukaryotic, bacterial or viral) acquired by lateral/horizontal gene transfer (Filée 2009). This led to the concept of ‘giant viruses are giant chimeras’ (Moreira and Brochier-Armanet 2008). The mimivirus was found to contain nearly all NCLDV core genes and clustered phylogenetically with phycodnaviruses based on these (Claverie et al. 2009, 2006). This suggested that it was an oversized NCLDV. Nonetheless, the translation system component genes seemed to illustrate a divergent evolutionary past. On the corresponding phylogenetic trees, mimiviruses clustered on a distinct branch, separated from the three established domains of cellular life (bacteria, archaea and eukaryotes). This striking novel observation has triggered the ‘fourth domain hypothesis’, according to which giant viruses evolved from cellular ancestors, most likely of an extinct fourth domain, via the reductive evolution route (Colson et al. 2012, 2011). However, the most recent evolutionary reconstruction favoured a less peculiar alternative hypothesis, the multiple origin of viral gigantism (Koonin and Yutin 2018). This latter analysis, based on well-conserved genes, defined three major branches on the NCLDV tree, suggesting at least three independent emergences from smaller viruses. For the translation-related genes, phylogenetic analysis showed kinship between viral and different eukaryotic lineages, suggesting lateral gene transfers at different time points of the virus evolution. Further evolutionary reconstructions revealed a connection between NCLDVs and smaller eukaryotic viruses, e.g. adenoviruses, and ultimately, derived all these viruses from tailless bacteriophages (Koonin and Yutin 2019). The reconstructed phylogeny was as follows. Branch #1 included most of the real giants (>500 kb): the extended family *Mimiviridae* and pandoraviruses. Branch #2 gathered the families *Asco-, Irido-*, *Marseille-* and *Pithoviridae*. This branch contained the widest variation of genome sizes (100 kb to 1500 kb). Branch #3 included only non-giants in two distinct clades: *Asfarviridae* (African swine fever virus and its protist-infecting relatives) and *Poxviridae*. According to the present knowledge, a major host switch took place two times along this branch. Once during the early history of poxviruses, which infect both arthropods and vertebrates, two animal phyla that radiated from the common ancestor more than 500 million years ago (Oliveira et al. 2017). Thereafter an apparent coevolution with the hosts also took place within the two subfamilies resulting in what we see today: several genera and poxvirus ‘types’ infecting a wide range of arthropod and vertebrate species. The second host switch happened at a later evolutionary stage in the sensu lato asfarviruses. Within this group a few apparently related viruses infect protists and pigs too (Alonso et al. 2018; Andreani et al. 2017; Bajrai et al. 2016).

Shifting the perspective to branch #2 families: we can see that using a concatenated set of 9 genes common to these, a close relationship has been confirmed, suggesting that ascoviruses emerged recently and share a common ancestor with invertebrate iridoviruses (Piégu et al. 2015). However, the replication strategies and morphologies are markedly different in these two virus groups replicating in invertebrate hosts (Federici et al. 2009). The evolutionary steps leading to these significant alterations still remain obscure.

This phylogenetic reconstruction of the NCLDVs demonstrated a ‘turbulent evolution’, which was dominated by gene gain, while on other branches substantial gene loss was apparent. The branches that include giant (protist) viruses are the most prominent gene gainers, whereas NCLDVs infecting animal hosts have undergone considerable gene losses, a sort of ‘genome contraction’ during their evolution from ancestral protist viruses. It was hypothesised that in animals, the selective pressure for virus genome size is stronger than in protists, yet its mechanism needs to be analysed (Koonin and Yutin 2018).

## Polyomaviruses

Polyomaviruses (PyVs) are non-enveloped icosahedral DNA tumour viruses with a double-stranded, cca. 5000-bp-long circular DNA genome. Approximately 80 accepted PyV species form the family *Polyomaviridae*; the species are clustered into four genera (*Alpha-, Beta-, Gamma-* and *Deltapolyomavirus*) according to the ICTV. The taxonomical classification of PyVs is based on the genetic distance of the large tumour antigen. Still, some PyVs cannot be categorised into any accepted genus, so these require additional genera in the future (Moens et al. 2017a).

The organisation of the circular PyV genome is highly conserved: the 5–7 genes are located on both strands, and distributed into early and late regions. There is a non-coding control region as well. The early coding region contains regulatory proteins: the small and the large tumour antigen. The late coding region contains the genes of the structural proteins, like the major (VP1) and two minor capsid proteins. The approximately 500-bp-long non-coding control region contains regulatory elements and transcription promoters, the origin of DNA replication (Moens et al. 2017b).

PyVs are known to infect mammals and birds, but using viral metagenomics, PyVs were identified and characterised also in fish and arthropods (Buck et al. 2016; Peretti et al. 2015). Analysing the available divergent sequence data of PyVs, results indicate that PyVs have been progressively coevolving with their hosts for about half billion years. Still, the current taxonomic classification system does not reflect this coevolution. This might be explained using phylogenetic analyses of PyV genes: some modern PyV species arose after ancient recombination events involving distantly related PyV lineages (Buck et al. 2016). The mammalian PyVs are known to be highly host specific and coevolving with their hosts. PyVs are generally apathogenic in humans, causing symptoms mainly in immunosuppressed individuals. However, e.g. Merkel cell polyomavirus (species *Human polyomavirus 5*) is oncogenic like several other mammalian PyVs.

Intra-host molecular evolution is also a feature of some PyV infections in humans. Mutations in the early region can lead to the expression of a truncated form of the large tumour antigen protein. Rearrangements in the non-coding control region and point mutations in VP1 have been described in two important human PyVs: JCPyV and BKPyV (Helle et al. 2017). In the case of JCPyV, mutations of these loci lead to the development of progressive multifocal leukoencephalopathy. Non-coding control region rearrangements in BKPyV are proposed to play a direct role in the development of PyV-associated nephropathy. Therefore, intra-host viral evolution appears to be an essential component of the disease process (McIlroy et al. 2019).

As discussed already, a recent host switch or a broader host range is usually associated with elevated pathogenicity. This is true for PyVs too: closely related PyVs can be detected in healthy bats of the genus *Rhinolophus* (Carr et al. 2017), whereas the goose haemorrhagic polyomavirus infects geese, Muscovy ducks and mulards, and the budgerigar fledgling disease virus (species *Aves polyomavirus 1*) infects birds of diverse families (Johne and Müller 2007).

## Circoviruses

Circoviruses (family *Circoviridae*) are vertebrate-infecting, single-stranded DNA (ssDNA) viruses with one of the smallest (~2 kb) virus genomes (Parrish 2011). The circular genome contains two genes (*rep* and *cap*) encoding the replication-associated (Rep) and the capsid (Cap) proteins, respectively (Biagini et al. 2011). The first recognised circoviruses, namely porcine circovirus 1 infecting swine and wild boar (Tischer et al. 1982, 1974) and the beak and feather disease virus of parrot species (Pass and Perry 1984) have been known for a long time. Still, the diversity of circoviruses and circular replication-associated protein encoding ssDNA (CRESS DNA) viruses was not revealed until recently. The continuous improvement of molecular methods has facilitated the discovery of numerous novel circo- and CRESS DNA viruses over the past few years (Delwart and Li 2012; Rosario et al. 2017; Shulman and Davidson 2017; Zhao et al. 2019).

Unfortunately, as most of this knowledge originates from metagenomic surveys of environmental samples, the exact host–virus linkage is often hard to decipher. In such cases, different comparative in silico approaches, like cophylogeny analysis of host and virus or a nucleotide composition analysis might help find a solution (Harrach 2000; Kapoor et al. 2010; Kemenesi et al. 2017; Shackelton et al. 2006). The results of some phylogenetic tree reconstructions suggested the potential long-term adaptation of some circovirus groups to birds, mammals, reptiles and fish (Altan et al. 2019; Delwart and Li 2012; Dennis et al. 2018; Fehér et al. 2013).

In relation to circoviruses and their hosts, the research of EVEs and their potential role in host cells has deepened recently. It was revealed that the complete or partial circovirus genome might be integrated into the host genome as an EVE. To date, primarily *rep*- but also *cap*-homologues have been detected in different animal genomes using bioinformatics approaches (Aiewsakun and Katzourakis 2015; Aswad and Katzourakis 2012; Belyi et al. 2010; Dennis et al. 2019, 2018; Fehér et al. 2013; Gibbs et al. 2006; Gilbert et al. 2014; Holmes 2011; Horie and Tomonaga 2011; Katzourakis and Gifford 2010; Krupovic and Forterre 2015; Liu et al. 2011). There are various possible functions—e.g. antiviral protection—proposed for different circoviral EVEs depending on the location and type of integration, but the exact impact of these is poorly known (Aswad and Katzourakis 2012; Dennis et al. 2018; Honda and Tomonaga 2016; Horie and Tomonaga 2011; Katzourakis and Gifford 2010; Liu et al. 2011). In the authors’ opinion, the quick evolution of these viruses via recombination (Lefeuvre et al. 2009; Martin et al. 2011; Rosario et al. 2012) and the RNA virus-like high mutation rates (Duffy et al. 2008; Firth et al. 2009; Rosario et al. 2012) can facilitate genomic integration, adaptation to the host cell or host switches. This hypothesis is supported by studies of related viruses (Gibbs and Weiller 1999).

By comparative analyses of the Rep-encoding gene superfamily, some authors propose the plant-infecting geminiviruses as the ancestors of both nanoviruses and circoviruses (Londoño et al. 2010; Mankertz et al. 1997; Meehan et al. 1997; Rosario et al. 2012). In the opinion of Gibbs and Weiller (1999), a plant-infecting nanovirus had switched to a herbivorous vertebrate first, and then recombined with a picorna-like virus (supposedly a calicivirus) via a retrotransposable element or a retrovirus. If we dig even deeper, the homologues of the ssDNA virus Rep were described in plasmids of eubacteria and algae, suggesting an evolutionary relationship (Gibbs et al. 2006; Liu et al. 2011; Oshima et al. 2001). In some authors’ opinion, the close phylogenetic relatedness of several circoviruses replicating in various host species may be the sign of cross-species transmissions (Delwart and Li 2012; Gibbs et al. 2006; Li et al. 2011; Liu et al. 2011). The common presence of the highly conserved Rep and the mechanism of the rolling circle replication (Faurez et al. 2009) are the most obvious evidences for the common ancestry of the ssDNA virus taxa and ssDNA molecules (Martin et al. 2011). Members of the family *Circoviridae* and related viruses have been existing for 40 to 50 million years. Some authors hypothesise even 100 million years of coevolution with the vertebrates based on EVEs (Belyi et al. 2010; Delwart and Li 2012; Katzourakis and Gifford 2010). By that period—from the middle Cretaceous to the late Eocene epoch—the main vertebrate groups had evolved already (Ravi and Venkatesh 2008). Others estimate the age of bird and mammalian circoviruses to be a mere 500 years, questioning long-term coevolution with the hosts (Firth et al. 2009). The evolutionary route of these viruses is not clear yet, and further research is needed both for circoviruses and their hosts.

## Concluding remarks

As seen from the example of this latest mentioned dispute concerning the appearance of circoviruses, there are a number of unsettled questions in connection with the coevolutionary processes observed in DNA viruses and their hosts. In the paragraphs above, we have listed these major processes. (1) Apparent speciation and coevolution of adenovirus lineages (genera) with the different vertebrate families. Here, host switches to new taxa were marked both with shift in the genome content (codon usage, A + T content) and more severe pathology in the non-coevolved hosts. (2) In connection with herpesviruses (HVs) a longer coevolutionary past was supposed, largely synchronous with the more divergent (vertebrate and invertebrate) host lineages. Integrated HV genomes in host chromosomes were demonstrated to provide new data about the genome content of ancient HVs and a new HVs lineage. Moreover, the discovery of a HV core gene homologue in tailed bacteriophages established the phylogenetic relatedness between two seemingly unrelated virus orders (*Herpesvirales, Caudovirales*) and broadened the perspective for the coevolutionary studies. (3) Concerning NCLDVs, we have discussed that many earlier unrelated virus families with large/giant capsid and genome sizes and even more divergent host range (from protists to vertebrates) can be phylogenetically linked based on a subset of core genes. This phylogenetic reconstruction demonstrated a ‘turbulent evolution’, which was dominated by gene gain and gene loss via horizontal gene transfer both from other viruses and from host genomes. (4) In connection with polyomaviruses (PyV) the recombination among PyV lineages generating novel virus species was also mentioned, and the importance of the intra-host molecular evolution was discussed in more detail. It was demonstrated that point mutations and rearrangements in non-coding control regions both can critically alter the pathogenic potential, immunogenicity and target organs of related PyVs of a single host species. (5) In the case of circoviruses (or more broadly CRESS DNA viruses), higher mutation rates and rolling circle type replication of their ssDNA, alongside with recombination events and integration into host genomes as well as presumed cross-species transmissions of distant hosts were simultaneously forming the coevolutionary processes.

The above list is extensive, yet not exhaustive. The presented examples cover a large portion of the animal DNA viruses, but many known and yet unknown families with potentially diverse coevolutionary strategies were not covered. One of these could be the recently discovered ‘adomaviruses’, which are apparently products of rampant gene exchange between adeno-, papilloma- and polyomaviruses and their ancient and more recent (fish) hosts (Welch et al. 2018). As mentioned in the introduction, protein structure based homology searches can reveal such similarities between poorly sequence-conserved proteins and unravel the otherwise hidden evolutionary connections of viruses and their hosts.

Viruses have always been around. Their parasitic lifestyle had an enormous driving effect on the evolution of all organisms, and thus life would not be the same without them. Future research will hopefully shed light on the exact origin and coevolutionary history of all viruses.
